# Sociocultural understanding of Tuberculosis and implications for care-seeking among adults in the province of Zambezia, Mozambique: Qualitative research

**DOI:** 10.1371/journal.pone.0289928

**Published:** 2024-01-18

**Authors:** Celso Give, Chrissie Morris, Joanna Murray, Benedita José, Raimundo Machava, Sonali Wayal

**Affiliations:** 1 Development Media International, Maputo, Mozambique; 2 Development Media International, London, United Kingdom; 3 National Tuberculosis Control Programme, Ministry of Health, Maputo, Mozambique; University of Sharjah, UNITED ARAB EMIRATES

## Abstract

**Introduction:**

Mozambique has a high burden of Tuberculosis (TB) with an incidence of 368 per 100,000 population in 2020, coupled with a low all-form TB detection rate. The COVID-19 pandemic has exacerbated delays in timely diagnosis and treatment of new TB cases. Promoting active TB case finding is a national priority in Mozambique. We conducted qualitative research to explore factors influencing TB testing in Zambezia province in Mozambique.

**Materials and methods:**

One-to-one, semi-structured, audio-recorded telephone interviews were conducted to explore TB-related knowledge, and barriers and facilitators to TB testing. A sample of two TB Program staff, two community providers of TB services, and 19 community members (10 women and 9 men) was recruited, with support from provincial government TB staff, from four districts in Zambezia with a high TB burden. Interviews were transcribed verbatim, and thematic analysis was conducted. The Mozambican National Bioethics Committee for Health approved the study protocol.

**Results:**

Our study highlights that knowledge about TB symptoms and its causes is low, which could delay timely TB testing. Sociocultural beliefs often implicate certain types of sexual activity and women as causes of TB symptoms; for example, having sex with a widow who has not been traditionally purified, or with a woman who has had an abortion. Therefore, people usually tend to first seek care from traditional healers instead of going to a health facility. Additionally, stigma associated with HIV and TB also delays care seeking. Gender-related disparities in TB care seeking were also evident.

**Conclusions:**

This study provides valuable insights into how healthcare seeking for TB is influenced by sociocultural understanding of symptoms and gender dynamics. Therefore, interventions to promote timely and appropriate care seeking for TB should be contextually tailored, culturally appropriate, and gender sensitive.

## Introduction

Tuberculosis (TB) remains a significant global health concern. In 2020, an estimated 9.9 million people developed TB for the first time and 1.5 million people died due to the disease [1]. The burden of TB in adult men (56%) was higher than among adult women (33%) and children (11%) [1]. A modelling study has estimated that TB deaths in low- and middle-income countries (LMICs) with a high burden of TB could increase by 20% between the years 2020–2024, due to the negative impact of COVID-19 on timely diagnosis and treatment of new TB cases [2]. Due to the COVID-19 pandemic, the number of people newly diagnosed and notified with TB fell from 7.1 million in 2019 to 5.8 million in 2020, and gaps in TB diagnosis and reporting were higher among men than women [1]. Most TB transmissions occur between the onset of cough and initiation of treatment. Reductions and delays in diagnosis and treatment could have a significant impact on both disease prognosis at an individual level and transmission within the community. TB case finding, notification, and treatment therefore continue to remain a global priority [3, 4].

In Mozambique, the absolute number of TB deaths between 2015 and 2020 reduced by 35%; however, in 2020, TB incidence (368/100,000) and TB-HIV co-infections (101/100,000) continued to be high along with drug-resistant TB [1]. Moreover, the notification rate of TB in all forms was 323/100,000 inhabitants (97,093 cases) [5]. Zambezia is the second largest province in central Mozambique with more than 5.1 million inhabitants [6]. It has a TB notification rate of 353/100,000 inhabitants (20,944 cases), with a high prevalence of drug-resistant TB [5]. Therefore, Zambezia is a high priority province for TB prevention and control, with an emphasis on enhancing case detection [7].

Traditionally in Mozambique, TB case detection, diagnosis and notification has predominantly relied on passive case finding (waiting for individuals to present to a health facility with TB-related symptoms), potentially leading to a significant gap between the actual number of people with TB, and those who present to the health facilities and are diagnosed and notified [8]. Thus, in recent years the Mozambican National Tuberculosis Program (NTP) has been promoting active TB case finding as a national priority [7, 9] in all health facilities via Finding TB cases Actively, Separating safely, and Treating effectively (FAST) strategy, and at the community level via community health workers. However, timely TB case detection in Mozambique is affected by various factors. A survey conducted among diagnosed TB patients attending a health facility for treatment in Southern Mozambique has shown delays in patients presenting to health facilities despite having symptoms, as well as health system delays (interval between patient’s first contact with the health care system and initiation of TB treatment) of approximately two months [10]. Factors such as farming responsibilities, visiting a traditional healer first, low TB knowledge, and coexistence of a chronic disease, could lead to delays in care-seeking among adult TB patients [10]. Literature reviews of studies performed in LMICs have also highlighted that factors contributing to patient delays in TB care-seeking can vary by sex [11, 12].

The slow rate at which patients with TB report to health facilities is a major limiting factor in global efforts to control TB [13]. Understanding what causes delays in TB care seeking and why, is important to develop effective interventions to promote timely TB testing and treatment seeking. Despite the high prevalence of TB in Zambezia province, no studies have been conducted to understand TB-related care-seeking behaviours there. Moreover, existing literature from Mozambique does not provide insights into if and why TB-care seeking behaviour varies by sex. Therefore, the aim of this study is to explore the contextual understanding of TB and care-seeking patterns for TB symptoms, and to explore if these vary by sex among adults in Zambezia.

## Materials and methods

### Study setting

Active case detection strategy has been implemented in all districts in Zambezia in all hospitals and health centers via FAST strategy and in the community it is implemented by the community health workers who are trained to offer TB testing and treatment services. This study was conducted between June-July 2020 in the districts of Milange, Mocuba, Ile, and the City of Quelimane in Zambezia province. These districts were chosen because they have a high prevalence of risk factors associated with TB such as mining areas (Milange), poverty, and people living with HIV (Milange, Mocuba, Ile and City of Quelimane) [14]. They have therefore been identified as priority districts for promoting TB case detection by the Ministry of Health in Mozambique (personal communication with Zambezia’s Provincial Health Directorate (DPS)).

### Study design and sampling approach

A qualitative study was conducted, comprising of semi-structured interviews with (i) community members, and (ii) key informants, namely health professionals working in the field of TB with the National TB Program and TB program in Zambezia, and community health workers (CHWs) who work with a non-governmental organization (NGO) named *Ajuda Do Povo para Povo* (ADPP) in Zambezia to provide TB testing services and health promotion in the community. Due to their experience of working with the communities for TB prevention and service provision, the key informants and CHWs were interviewed to understand their perspectives regarding the attitudes of community members towards TB and the barriers and facilitators for TB testing. Insights from these interviews helped us to refine the topic guide we used for data collection with the community members. Topic guides were piloted with two participants and minor changes were made to enhance clarity.

To recruit community members, convenience sampling, a method commonly used in qualitative studies, was used [15, 16]. Community members were identified with the help of experts working with DPS and three community-based radio stations in Zambezia because they have frequent contact with community members and local leaders. Participants aged between 20 to 50 years, an age group at greater risk of TB [17], were eligible for recruitment. From the above-mentioned districts, names, telephone numbers and preferred contact times were collected by the experts from all potential participants who were interested in participating in the study. They were informed that a researcher would contact them regarding study participation. Additionally, a snowball method was used to ensure recruitment of community members from remote rural areas. To this end, at the end of each interview, the researcher asked the participant about their willingness to refer people from their social network (i.e., friends, neighbours, family etc.) located in remote areas of the study districts, to participate in the study. If a participant was willing to refer someone, they were asked to provide that person’s contact details and inform them that they will be contacted by a researcher regarding study participation. There was no obligation for participants to make a referral. To minimize recruitment bias, each participant was asked to provide details of only two contacts who were willing to be interviewed. Eight participants were recruited via snowball method. A purposive sample across age groups, by sex, and districts was planned to enable us to detect differences across these. We recruited community members until there were no new themes emerging from the data being collected.

To recruit the key informants, a study co-author (BJ), who is a member of staff at the NTP, was contacted directly by a researcher and interviewed in person. She introduced the researcher to a staff member from the DPS in Zambezia who was also interviewed in person. A coordinator of an NGO asked the CHWs, who work with them, about their interest in participating in the study prior to sharing their name and telephone details with a researcher. Subsequently, the researcher directly contacted the CHWs via phone.

Due to the risk of COVID-19 transmission, there were travel restrictions in place in Mozambique during the time of this study. Therefore, all the data collection with CHWs and community members was conducted via telephone by an experienced qualitative researcher (CG) who is an anthropologist and contacted all the potential participants and confirmed their eligibility to participate. Prior to data collection, the researcher read the consent form to all the potential participants to explain the study objectives and procedures, voluntary participation in the study, and their right to decline to participate in the study without facing any negative consequences. Subsequently, oral consent for study participation as well as for audio recording from each participant was obtained via telephone and audio-recorded as proof of consent to study participation. In-person as well as the phone interviews lasted for approximately 35 to 50 minutes. The Mozambican National Bioethics Committee for Health approved the study protocol (Ref:259/CNBS/20).

Participants were given the choice to be interviewed in local languages spoken in Zambezia or in Portuguese. All the participants (23) who were approached to participate in the study agreed to participate and opted to be interviewed in Portuguese. A topic guide was used to explore attitudes and perceptions of key informants towards people’s knowledge and care seeking behaviors for TB, especially TB testing, and any differences in these by sex. A separate topic guide, informed by data collected from the key informants, was used to explore community members’ perceptions about the causes of TB, its symptoms, and modes of transmission; awareness, barriers, and facilitators for TB testing and treatment; and attitudes towards people diagnosed with TB.

### Analysis

**Fig 1:**
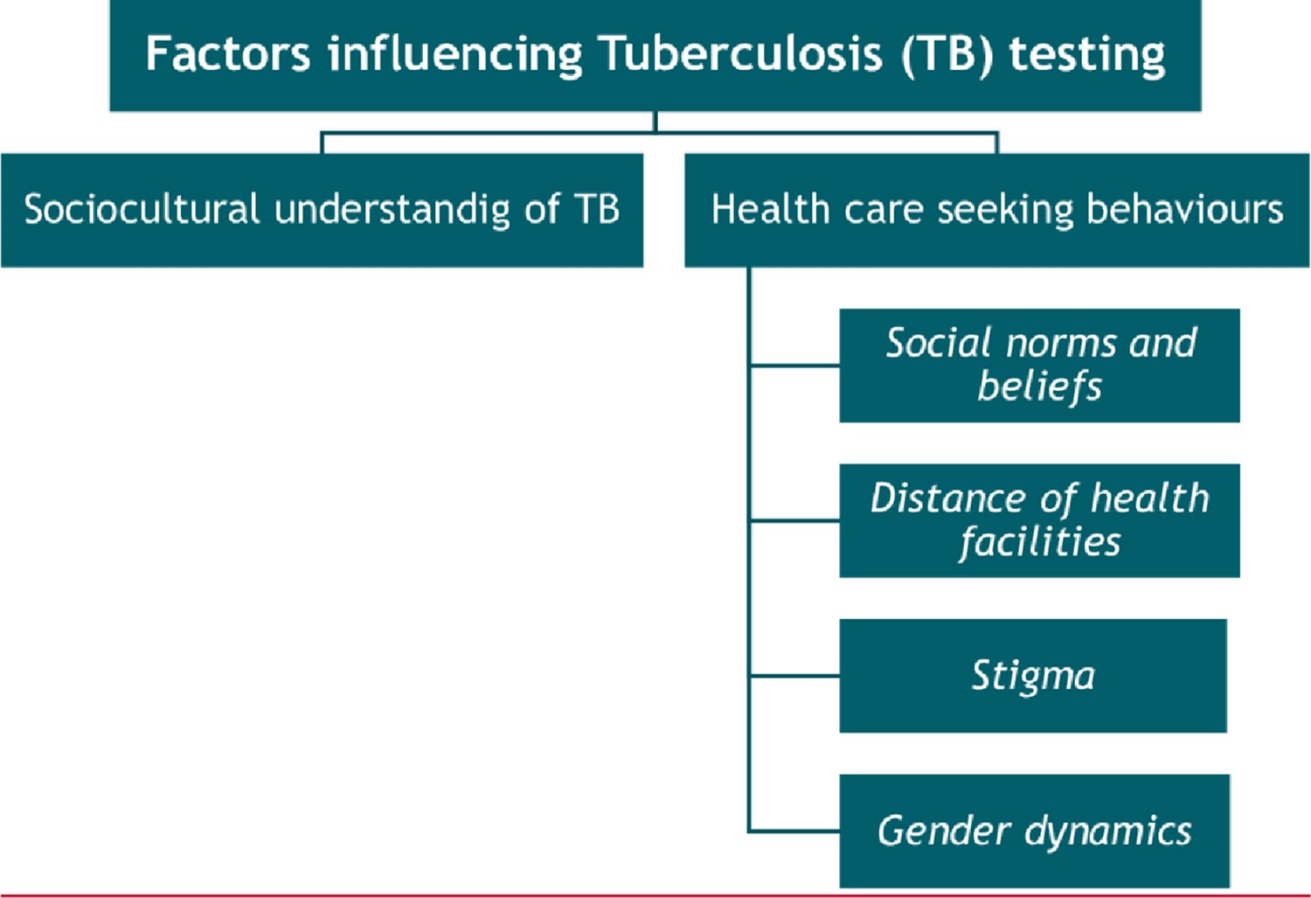
Factors influencing Tuberculosis testing.

All interviews were transcribed in Portuguese and a thematic analysis was conducted [18]. Initially a researcher (CG) read the transcripts verbatim, thoroughly, and repeatedly to familiarize with the data. Initially six transcripts were coded inductively by CG manually. Excel spreadsheet (S1 Data) was used to identify and extract data for the broad themes of interest explored during the interviews. Subsequently, data under each theme was analysed and codes were developed (See ***Fig 1***). These codes were then discussed by the research team (SW and CG) and revised if necessary to develop a codebook. Subsequently, all the transcripts were coded (CG) using the codebook while being mindful of new emerging codes. Data under each code was grouped together to identify emerging patterns and themes in an iterative manner. Transcripts were re-read to verify the accuracy of themes and sub-themes identified. To ensure confidentiality of the study participants and to account for the potential risk of stigma associated with sharing personal perspectives/experiences about TB, anonymised quotations illustrating participants’ own, or their perception of others’ knowledge, beliefs, experiences, and behaviours have been used. No interviewees participated in the discussions related to data analysis.

## Results

Twenty-three semi-structured interviews (4 with key informants and 19 with community members) were conducted. As shown in Table 1, more interviews were conducted with community members from Cidade de Quelimane because it has the highest TB prevalence in Zambezia. Similar numbers of men and women participated in the interviews. Overall, eleven and eight participants were recruited from urban and rural areas respectively. The results focus on the community members beliefs and perceptions about TB and healthcare seeking behaviors.

**Table 1:** Site of recruitment and socio-demographic characteristics of participants.

Characteristics	Health professionals/ community health workers (n = 4)	Community members (n = 19)
**Districts**		
*Cidade de Quelimane*	2	8
*Mocuba*	1	4
*Milange*	1	4
*Ile*	-	3
**Gender**		
*Female*	1	10
*Male*	3	9
**Age**		
*20–30*	-	7
*31–40*	4	9
*41–50*	-	3

### Socio-cultural understanding of Tuberculosis

Very few participants associated symptoms such as coughing, blood in sputum, loss of weight/appetite, etc with TB. Participants recruited from urban areas were more aware of TB symptoms than those from rural areas. This was reported to be due to greater exposure to TB-related information on the radio, via pamphlets in health centres, and relatively easy access to health centres in urban areas. Both male and female in rural areas lacked awareness about TB symptoms.

*I think we have a lot of information about tuberculosis*, *on the radio people talk about TB*, *some lectures are given about TB and if you go to the health units you will see that all the places have pamphlets about TB which promote TB testing if anyone had a cough and sputum*. (Male, 27 years old)

*In principle*, *when a person has a cough*, *they should go to the hospital*. *And there at the hospital*, *they do an analysis*. *They will see if the cough is persistent or not*, *give the medication*. *Now if you continue to cough*, *go back and do the analysis and that analysis decides whether you have it or not*. (Female, 35 years old)

However, most of the participants, both male and female, from urban as well as rural areas, perceived symptoms such as persistent cough, weight loss, etc., were caused by witchcraft, adultery, or sexual involvement with “impure women”. Women who had lost their husband and had not yet performed purification rituals or women who had had an abortion were perceived to be “impure women”.

*Many people in my community think that tuberculosis results from sexual involvement with another person’s wife or with impure women*. *When a woman loses her husband*, *she has to go to the traditional healer to perform purifications rituals*, *if she does not perform this ritual*, *she is perceived as impure*. (Male, 46 years old)

*I know a person who had tuberculosis and thought it was witchcraft because he had a good life and people were jealous of his life*, *so they cast a spell for him*. *There are a lot of people in the communities who think like that about tuberculosis*. (Female, 42 years old*)*

### Health care-seeking behaviours

Several factors influence healthcare seeking behaviour among people with TB symptoms.

#### Social norms and beliefs

Due to the above-mentioned perceptions and beliefs about the causes of TB symptoms, most of the participants mentioned that people usually seek help from traditional healers first, especially if the symptoms are perceived to be caused due to witchcraft or lack of performing purification rituals. Some participants felt that in general in Mozambique people usually seek help from traditional healers first. However, if their health condition does not improve, then people go to health facilities.

*If you grew up in a society where everything is treated on a traditional (healer) level*, *and even if you know that it can be cured in two days if you go to a health center*, *the person still goes to a traditional healer*. (Male, 41 years old)

Some participants felt that people seek care from traditional healers because they are perceived to be caring towards their clients and provide better health services compared to the health professionals in the health facilities. Some participants also felt that people seek care from traditional healers because healthcare provided at the medical health facilities is expensive, and health professionals do not offer timely TB testing.

*Traditional healers serve better than hospitals*. *When you go to the traditional healer you are welcomed*, *they talk with you and try to understand what is going on*. *At the hospital*, *they yell at you*. (Female, 37 years old)

*I was sick for a long time*, *then I went to the hospital*, *and they didn’t know what I had*. *For some time*, *I couldn’t even walk*, *I had to take other people’s help to be able to walk*. *I was getting frustrated*, *and I didn’t know what to do anymore*. *I was desperate and then they tested me at the hospital where they found out I had tuberculosis*. *This happens to many people*, *getting sick and not knowing what they have*, *a lot of people don’t know much about tuberculosis*. (Female, 33 years old)

#### Distance to health facilities

Participants also mentioned that people usually seek help from traditional healers first, especially in rural areas, because health facilities are far away. This makes accessing care expensive due to travel costs as well as opportunity costs (taking time away from work, especially for men; household chores and childcare for women).

*It is a long way from the communities to the hospital*, *if you do not have transportation to go there*, *you have no way to resolve it*. *Imagine that sometimes you have to walk more than 25 kilometers when you are sick*. *How can you do that*? *People end up going to the healer*, *it is closer and some like to go there*. (Female, 27 years)

#### Stigma associated with Tuberculosis and HIV

Some community members as well as key informants felt that the perceived association between TB and HIV/AIDS in the community is common. This negatively affects people’s healthcare seeking behaviour if they have symptoms of TB. People who live with others, fear being discriminated against due to the fear of TB transmission to other people.

*Currently*, *because of what is happening in society*, *TB is one of the diseases*, *the one most associated with HIV-AIDS*. *So*, *when people start to have these symptoms*, *they are a little more afraid of approaching hospitals*. *That ends up generating some fear due to the stigma that is still associated with HIV-AIDS*. *So*, *I think that when there are these kinds of symptoms*, *there are people who tend to hide their symptoms*. (Male, 38 years)

*Unfortunately*, *in the community*, *discrimination against people with TB persists*. *During our mobilization meetings and working in the communities*, *we can see that people are discriminated against due to the association of TB with HIV*. (Male CHW, 36 years)

#### Gender dynamics

Some female participants mentioned that women have limited access to health care, including if they have TB symptoms, because they lack decision-making power about where and when to seek health care. These decisions are usually made by spouses or senior male members of the family. Their financial dependence on men for transport and associated costs to go to the health facilities, further affects their decision-making capacity with regards to healthcare seeking.

*I met a lady who was sick in the hospital*. *She told me that she had told her husband that she did not feel well and that she should go to the hospital*. *Her husband denied it*, *but she went to the hospital and told him that she had tuberculosis and that she was going to start treatment and she would have to stay away from him for 6 months*. *The husband told her that if she wants to take the treatment*, *he will not be waiting for her until the treatment is over*. *She had to decide whether to lose her husband or undergo treatment*, *that is*, *to choose between her life and her husband*. *She decided to undergo the treatment and her husband left her*. *Now she is fine*. *Imagine if she had not had the treatment*. (Female, 38 years old)

Some male participants felt that health services are more targeted at women and so men have limited access to health care and feel excluded from services:

*I think that women have more advantages because they are the first to benefit from any type of hospital intervention compared to men*. *Not only that*, *even in the health system itself*, *there is now more attention focused on maternal and child health*. *So*, *men feel left out*. *So*, *they may be one of the last to be seen at the health center*. (Male, 36 years old)

## Discussion

This study explored factors influencing health care seeking behaviours for TB in Central Mozambique. Our study findings highlight that sociocultural beliefs and understanding of the causes and symptoms of TB contribute to delays in seeking timely and appropriate testing and treatment for TB. Similar to findings from studies conducted in other African countries, TB symptoms were perceived to be a manifestation of witchcraft [19, 20]. As reported in a study conducted in Southern Mozambique [21], TB symptoms were also perceived to be caused due to transgression of sexual or social norms and customs, and were associated with stigma. For example: sexual engagement with people perceived to be impure, or not performing a purification ceremony following a death in the family. Sex was perceived to be a mode of TB transmission [16]. This probably led to community members sharing their experiences and beliefs in third person. Women were often perceived to be impure, either due to the death of their husband or undergoing an abortion. Such rationalization of TB symptoms leads to women being blamed for the illness. Anthropological studies have shown that such notions of impurity as a cause of illness are embedded in the broader belief system in Mozambique and other African countries, and such illnesses are perceived to be contagious [22]. Nevertheless, sometimes the sociocultural interpretations of causes of TB co-existed with bio-medical understanding of the illness, usually in the urban areas [23].

Our findings suggest that such sociocultural interpretations of the causes of TB typically encourages people to consult traditional healers in times of distress and ill-health to identify the source of the problem, to eliminate the source of the evil, and to avoid it in the future [24]. Similar to findings from a study conducted in Uganda, our study found that traditional healers were preferred to healthcare providers because they were perceived to be respectful and supportive compared to the health professionals, indicating that healthcare users value the importance of interpersonal interactions and trustworthiness of healers [25]. Moreover, as reported in studies done in other African countries, perceived stigma associated with TB and the association of TB with HIV negatively affects timely care seeking [19, 26]. Poor knowledge and stigma associated with TB could lead to diagnostic delays [27], delays in appropriate care seeking [11, 21] and influence where people seek care in Mozambique. Health literacy is an important predictor of health status [28], thus improving TB related knowledge in Mozambique is vital. However, these data highlight that interventions promoting TB-related health literacy should be contextually tailored and culturally appropriate.

However, unlike a survey conducted in Ethiopia [19], our study did not find differences in knowledge about TB among men and women. Nevertheless, gender norms influenced TB care seeking. Delayed healthcare seeking among men was due to work pressures, and feelings of alienation from health care facilities which were perceived to be places for women and children, and as being insensitive towards men’s healthcare needs [29]. Whereas among women, access to TB health care services was affected by the demands of childcare and household chores made on women, as well as by their limited decision-making power and financial dependence on their spouse or other family members. Therefore interventions promoting knowledge and awareness of TB in Mozambique should be gender-sensitive, and should be accompanied by TB services which are tailored to the gender-specific barriers to accessing services [12].

Ending the TB epidemic by 2030 is a Sustainable Development Goal [28]. However, to address the threat of TB, innovative approaches that extend beyond clinics and hospitals are needed [13]. A study conducted among women in India has shown that participants who had seen TB campaigns on any mass media had better knowledge about TB than those who had not [30]. A mass media health education campaign in Cali, Colombia showed that providing basic information about TB symptoms and the procedures for diagnosis can increase diagnostic coverage [30]. Increasing public awareness of TB disease could potentially promote voluntary reporting to health facilities for TB care and reduce stigma towards TB patients [16, 29]. Therefore, we used data from this research to develop a mass media campaign to promote awareness of TB symptoms and promote testing for TB in Zambezia.

Our study has contributed to the limited existing literature on factors influencing care seeking for TB symptoms in Mozambique. However, the study findings should be interpreted considering its limitations. This study was conducted during the state of emergency declared by the President of Mozambique in March 2020 due to Covid-19, which led to the research being conducted remotely via telephone. This meant that there was no observation of nonverbal cues of the participants during the interviews which may have affected the nuances of the data collected. The recruitment of study participants was done by staff of community radio stations and the DPS which may have introduced some selection bias. However, we tried to minimize this bias by using a snowball approach to recruit community members from participants’ social networks from remote areas in Zambezia. Lastly, the study was conducted in the province of Zambezia, limiting the generalizability of the study findings to the whole country. However, the significant overlap between our study findings and those of a study conducted in Southern Mozambique [19] indicates that some perceptions about symptoms and causes of TB, and barriers to TB care seeking are likely to be generalizable beyond the study area. Further studies should explore TB related care seeking behaviors and knowledge in other parts of Mozambique, which continue to have high TB prevalence.

## Conclusions

Our study provides valuable insights into how healthcare seeking for TB is influenced by sociocultural understanding of symptoms and gender dynamics. Therefore, interventions to promote timely and appropriate care seeking for TB should be contextually tailored, culturally appropriate and gender sensitive.

## Supporting information

S1 Data Example of coding raw data by theme. (DOCX)

S1 File Topic guides. (DOCX)
