# Supplementary material for: Sociocultural understanding of Tuberculosis and implications for care-seeking among adults in the province of Zambezia, Mozambique: Qualitative research
Source: PLoS One. 2024 Jan 18;19(1):e0289928. doi: 10.1371/journal.pone.0289928 (PMC10795997; doi:10.1371/journal.pone.0289928)
Supplement: S1 Data — (DOCX) [file pone.0289928.s001.docx]

**S 1. Example of coding raw data by theme**

| **Participant ID** | **Understanding of Tuberculosis: biomedical** | **Understanding of Tuberculosis: sex** | **Understanding of Tuberculosis: cultural** | **Health care-seeking behaviours: distance to health facilities** | **Health care-seeking behaviours: Stigma** | **Health care-seeking behaviours: social norms** | **Health care-seeking behaviours: gender** |
| --- | --- | --- | --- | --- | --- | --- | --- |
| EMC_ F_28 years old | TB is a contagious disease caused by a bacillus, but it has treatment |  | When it comes to communities living in remote (rural) areas, tuberculosis is not very well known. When the person gets sick, they start with symptoms, they look for a healer, then they only look for a hospital later. Many lose their lives to TB because they think it is caused by witchcraft. | I think that people at some point have had difficulties going to the hospital to seek medical assistance because the hospital is far away | in communities, as some do not know much about tuberculosis, they tend to think that they have HIV and, as they are pointed out (stigmatized) by others, they tend to avoid treatment for fear of talking about them. | But as we are Africans, other people often go to *curanderos* (traditional healers) to be cured of this disease. I have information that at the healer people are given many herbs and roots to take and pay some money and others pay a chicken or scapula….this depends on each healer. | As a woman, I think that women are capable of seeking treatment when they have symptoms of tuberculosis. But some husbands prevent their wives from doing this (seeking treatment at the hospital). |
| MC, Male, 32 years old | For me if someone had a cough for more than 7 days I could advise him to go to the hospital to make an appointment and find out about his health. For me, when people suspect they have tuberculosis, they go to the hospital to see a nurse to do the analysis. | They say that tuberculosis is a disease that you catch when you have (sexual) intercourse with a person who had an abortion | There are people who still believe that TB is a traditional disease. | long distances to hospitals and lack of transport, people in these communities also do not like to get used to the hospital. |  | My cousin when she had tuberculosis told me she went to the healer and was given some herbs and roots to take. | In my view, women are the ones who have get more health care more than men. |
| MC_Female_28 years old | I just know that when you arrive at the hospital, people are tested for tuberculosis. They are collected Spittle to do the test. This is done in the hospital laboratory. | Here in the community, they usually say that when a woman loses her husband, she must perform the *Khopacha* rituals (calling the ancestors for the purification of the person). When you don't do *Khopacha* and have sex with someone else *,* you get tuberculosis | Here in the community, they usually say that when a woman loses her husband, she must perform the *Khopacha* rituals (calling the ancestors for the purification of the person). When you don't do *Khopacha* and have sex with someone else*,* you get tuberculosis | Before there were difficulties with the health centres being far away, but now it has improved. | One of the barriers is always thinking that it is associated with HIV and feeling ashamed to go to the health centre |  | For me, there is no difference between women and men in seeking these treatments in hospitals, as everyone is concerned about their health status. |
| MC_Woman_36 years old | Any exposed person can contract TB, when being in a closed environment with someone who has TB, being closer. When I was teaching, I had someone who contracted TB and the whole family contracted TB, including the children. To say that there was no care taken to prevent the person from passing on TB to others. | It is a traditional practice to seek traditional treatment for such symptoms if one had an abortion, or was involved with an impure woman. | people usually say that when someone gets TB it's because there was a traditional fault or family problems (when they don't respect their ancestors). | Distance and service at the health facility is slow. It has been luck, it has taken a month to analyze the urine and saliva tests, it would be good if they implemented a quick test like the HIV test. Imagine someone who can't spend 20mt a day and being told to come back tomorrow, next week, that makes people despair. And the distance to the health facility is too great. | I remember that when I had tuberculosis I was very thin and I told my husband to go to the hospital, but he didn't accept it. People in the community said that I have AIDS (HIV) because I was very thin and I coughed all the time. This way of seeing people in the community makes other people afraid of taking the medication, there is a lot of discrimination in the community. Tuberculosis is AIDS for them | In a first phase they (people) look for traditional care (they go to healers) they receive herbs and roots, but with the education that exists now, people also look for hospital care. |  |
