# Supplementary material for: Sociocultural understanding of Tuberculosis and implications for care-seeking among adults in the province of Zambezia, Mozambique: Qualitative research
Source: PLoS One. 2024 Jan 18;19(1):e0289928. doi: 10.1371/journal.pone.0289928 (PMC10795997; doi:10.1371/journal.pone.0289928)
Supplement: S1 File — (DOCX) [file pone.0289928.s002.docx]

1. ***Guiões de entrevistas semiestruturadas com membros da comunidade e informantes-chave***

#### Guião de entrevista semiestruturada com membros da comunidade

Este guião será usado para explorar o entendimento sociocultural sobre a tuberculose (TB), atitudes e crenças relacionadas à barreiras, facilitadores e comportamentos na busca de cuidados de saúde para a TB.

**Informação do participante**

| Sexo Masculino ______. Feminino______  Idade: ________  Local: _________  Estado Civil: ______ |
| --- |

1**. Entendimento sociocultural, atitudes e crenças na busca de cuidados e serviços para a tuberculose**

a) Qual é a doença mais comum na sua comunidade? Você sabe porquê razão as pessoas apanham mais essa doença?

b) Você já ouviu falar da tuberculose? (use alguns termos locais que podem ser usados para TB se as pessoas não entenderem) Explore:

c) O que é TB?

d) Como as pessoas podem saber se têm TB?

Explora: a compreensão das pessoas sobre sintomas e sinais de TB

e) Quem você acha que pode contrair tuberculose?

f) Por que você acha que algumas pessoas contraem TB?

Explore: compreensão sociocultural das pessoas sobre a tuberculose; explorar especificamente percepções sobre se as mulheres contraem tuberculose e porquê; e percepções de como a tuberculose é transmitida.

**2. Contexto de procura de cuidados de saúde**

a. Se alguém tiver tosse por mais de 7 dias, o que você recomendaria que fizesse? Porquê?

b. Você sabe o que as pessoas fazem se acharem que têm TB? Explora:

• se os participantes pensam que as pessoas não fazem nada, pergunte porquê?

• Se os participantes pensam que as pessoas procuram atendimento para a tuberculose, pergunte, normalmente quando as pessoas procuram atendimento (após quantos dias de sintomas como tosse, cansaço, expectoração esverdeada/amarelada); onde as pessoas costumam procurar atendimento (por exemplo, curandeiros, mercados, lojas, agentes comunitários de saúde, centro de saúde: se eles forem a mais de um local, então procure saber, onde as pessoas tendem a procurar primeiro os serviços de saúde e por quê), que tipo de cuidados de saúde as pessoas recebem nesses locais (ervas, rituais, fazem testes (peça para descrever como), tomam comprimidos etc.), eles têm que pagar para procurar atendimento? como é feito o pagamento (dinheiro, espécie etc)?

• Pergunte se existem diferenças na busca de cuidados e serviços de saúde para TB entre Homens e Mulheres.

• As pessoas podem procurar assistência médica facilmente? Caso contrário, verifique as razões (transporte, custo, percepções sobre cuidados médicos, etc.).

• As mulheres são capazes de buscar tratamentos quando têm sintomas da TB? Se não, porquê? As mulheres enfrentam os mesmos problemas (obstáculos) que os homens para busca de tratamento para TB? Se as mulheres buscam atendimento, o que elas costumam fazer?

c. Existe um agente comunitário de saúde na sua área? Explore:

• Se eles fornecem serviços para TB? Que tipo de serviços eles fornecem?

• As pessoas usam os serviços fornecidos pelos agentes comunitários de saúde para TB? Se sim, para que fins eles utilizam os serviços dos agentes comunitários de saúde? Se não, porquê?

d. Você sabe se as pessoas vão aos centros de saúde para procurar cuidados para TB? Explore: Se não, porquê? Se sim, pergunte para onde eles vão? O que acha que acontece quando uma pessoa com sintomas de TB vai ao centro de saúde (peça que descreva a sua compreensão do que acontece lá) Explore: Como os profissionais de saúde tratam as pessoas que têm TB?

**3. Conhecimento, atitudes e crenças sobre o teste da TB**

Você sabe como as pessoas podem fazer o teste da TB? Explore:

• Explorar suas percepções sobre como as pessoas podem ser testadas?

• se eles sabem sobre exames e coleta de escarro, radiografia de tórax?

• Explorar seus pontos de vista sobre o processo de coleta de amostras para teste da TB

Você acha que as pessoas devem fazer o teste de TB? Se sim, onde eles fazem a testagem para a tuberculose? Se não, por que as pessoas não testam para a tuberculose?

**4. Atitudes, crenças e conhecimento sobre o tratamento da TB**

a) Você acha que a tuberculose pode ser tratada? Se não, porquê? Se sim, como?

b) Onde é que as pessoas podem obter medicamentos para a TB? (explore os desafios que as pessoas podem ter para obter os medicamentos/comprimidos)

**5. Conclusões**

a) O que pode ser feito para que pessoas a procurarem mais cuidados e serviços para a tuberculose? Peça exemplos.

b) Gostaria de saber se você tem alguma coisa adicional relacionadas com à tuberculose que não abordamos durante a nossa conversa e que gostaria de partilhar?

Muito obrigado pelo seu tempo

#### Guião de entrevistas semiestruturadas com informantes-chave

**Informação do participante**

| Sexo Masculino ______. Feminino______  Idade: ________  Local: _________  Estado Civil: ______ |
| --- |

1. Você acha que as pessoas têm conhecimento sobre a tuberculose? Eles reconhecem os sintomas da tuberculose?

2. Você acha que as pessoas procuram cuidados quando têm sintomas da TB? Se não, porquê? Se sim, onde acha que as pessoas procuram atendimento/cuidados? Isso varia de acordo com o sexo?

3. O que acha que leva alguns pacientes demorarem procurar cuidados/atendimento para os sintomas da TB? Isso varia de acordo com o sexo?

4. Você acha que as pessoas sabem onde testar para a tuberculose? Isso varia de acordo com o sexo?

a. Existem desafios na coleta de amostras para TB?

b. Existem outros desafios na testagem para pessoas com TB? Isso varia de acordo com o sexo?

c. Existem desafios no diagnóstico clínico da TB? Isso varia de acordo com o sexo?

5. Existem desafios no tratamento da TB? Eles (os desafios) variam por sexo?

6. O que você acha que pode ser feito para melhorar a detecção de casos de TB?

7. Existe algum custo que as pessoas tenham que encorrer pela procura de tratamento para TB? (Explore: financeira, relacionada a viagens, tempo necessário para obter exames e tratamento da TB)

8. Tem mais alguma coisa adicional relacionada a procura de cuidados de saúde para tuberculose que durante a conversa não abordamos e que gostaria de partilhar?

Muito obrigado pelo seu tempo.
